# Amygdala functional connectivity is associated with social impairments in preterm born young adults^☆^

**DOI:** 10.1016/j.nicl.2018.101626

**Published:** 2018-12-03

**Authors:** Christina B. Johns, Cheryl Lacadie, Betty Vohr, Laura R. Ment, Dustin Scheinost

**Affiliations:** a Yale School of Medicine, New Haven, CT, USA; b Department of Radiology and Biomedical Imaging, Yale School of Medicine, New Haven, CT, USA; c Department of Pediatrics, Warren Alpert Medical School of Brown University, Providence, RI, USA; d Department of Pediatrics, Yale School of Medicine, New Haven, CT, USA; e Department of Neurology, Yale School of Medicine, New Haven, CT, USA; f Child Study Center, Yale School of Medicine, New Haven, CT, USA

**Keywords:** Amygdala, Magnetic resonance imaging, Resting-state, Social development, Preterm birth

## Abstract

Survivors of preterm birth experience long-lasting behavioral problems characterized by increased risk of depression, anxiety, and impairments in social functioning. The amygdala is a key region for social functioning and alterations in amygdala structure and connectivity are thought to underlie social functioning deficits in many disorders, including preterm birth. However, functional connectivity of the amygdala and its association with social impairments is not well-studied in preterm participants (PTs). In a group of late adolescents born very PT (600–1250 g birth weight), measures of social and emotional development were examined using the Child Behavior Checklist (CBCL) administered at age 16 (66 term and 161 preterm participants), the Youth Self Report (YSR) administered at age 16 (56 term and 45 preterm participants), and the Vineland Adaptive Behavior Scales (VABS) administered at age 18 (71 term and 190 preterm participants). Amygdala functional connectivity was also examined using resting-state functional magnetic resonance imaging at age 20 (17 term and 19 preterm participants). By parent report, preterm-born adolescents demonstrate increased social impairment compared to their term-born peers. Amygdala connectivity is altered for those prematurely-born, and markers of social functioning correlate with altered amygdala-PCC connectivity. These findings add to knowledge regarding the developmental trajectory of amygdala connectivity in PT and suggest a possible neural underpinning for the well-characterized social impairment experienced by prematurely-born individuals.

## Highlights

• By parent report, preterm adolescents demonstrate increased social impairment.
• By self-report, preterm adolescents demonstrate no social impairment.
• Amygdalar connectivity is altered for those preterm young adults.
• Markers of social functioning correlate with altered amygdala-PCC connectivity.

## 1 Introduction

Preterm (PT) birth is a significant global public health problem with as many as 11% of global live births occurring before 37 weeks of gestation.(Blencowe et al., 2013) Survivors of preterm birth experience long-lasting behavioral problems characterized by increased risk for depression, anxiety, and impairments in social functioning.(Bhutta et al., 2002; Eryigit-Madzwamuse et al., 2015; Fenoglio et al., 2017; Healy et al., 2013; Johnson and Marlow, 2014; Montagna and Nosarti, 2016) These symptoms present early in childhood and last into adulthood, with increased internalizing behavior, impaired emotional regulation, and poorer peer play in early childhood(Boyd et al., 2013; Jones et al., 2013; Spittle et al., 2009) and with impaired social adjustment and increased risk for bullying in adolescence.(Allin et al., 2006; Yau et al., 2013) Even in adulthood, PT are less extroverted, take fewer risks, and have lower self-esteem compared to their term-born peers.(Eryigit-Madzwamuse et al., 2015; Saigal et al., 2016b) Because of these impairments in social functioning, PT-born adults are less likely to maintain committed relationships or become parents.(D'Onofrio et al., 2013) In addition, these symptoms have been linked to increased psychiatric morbidity in the PT population at young adulthood, including anxiety, depression, and social phobias. (Burnett et al., 2011; Healy et al., 2013; Johnson and Marlow, 2011, Johnson and Marlow, 2014; Van Lieshout et al., 2015).

A key brain region for social functioning is the amygdala.(Adolphs, 2009) Lesion studies show that damage to the amygdala impairs individuals' abilities to recognize complex social emotions in facial expressions.(Adolphs et al., 2002; Shaw et al., 2005) Amygdala volume and functional connectivity with cortical regions correlates with social network size in young adults,(Bickart et al., 2012; Bickart et al., 2011) and alterations to amygdala circuitry contribute to social processing deficits in many disorders, such as autism spectrum and anxiety disorders.(Baron-Cohen et al., 2000; Bickart et al., 2014; Kim et al., 2011) Similarly, reduced social functioning in PTs has been attributed to alterations in amygdala structure and function.(Cismaru et al., 2016; Fenoglio et al., 2017; Papini et al., 2016; Rogers et al., 2017)

While alterations in functional connectivity for specific networks, such as language, are well characterized across development in those prematurely born, (Kwon et al., 2016) functional connectivity of the amygdala has been less well-studied. In PT neonates, amygdala connectivity is decreased to frontal cortex and sub-cortical regions(Rogers et al., 2017; Scheinost et al., 2016) and correlates with internalizing symptoms at 2 years of age (Rogers et al., 2017). In PT adults at 30 years of age, amygdala connectivity is decreased to the right posterior cingulate cortex, left precuneus, and increased to the superior temporal sulcus (Papini et al., 2016). While amygdala connectivity exhibits large-scale changes during adolescence/young adulthood in typically developing controls, (Gabard-Durnam et al., 2014; Gee et al., 2013) this age range has not been examined in previous studies of amygdala connectivity in PTs. Together, these studies suggest the need to investigate the association between social functioning and amygdala connectivity in PT adolescents.

In this work, social functioning and amygdala connectivity during late adolescence in a cohort of very PT and term control participants were examined. Measures of social and emotional development were evaluated by both parent and self report at ages 16 and 18. Assessment scores were then compared to amygdala functional connectivity using resting-state functional magnetic resonance imaging (fMRI) between study groups at age 20, and finally, individual differences in social behavior were correlated with alterations in the amygdala. It was hypothesized that PTs would demonstrate decreased social functioning in late adolescence when compared to their term-born peers, and that impaired social functioning would correlate with altered amygdala connectivity.

## 2 Methods

This study was performed at the Yale University School of Medicine in New Haven, CT, the Warren Alpert Medical School of Brown University in Providence, RI, and Maine Medical Center in Portland, ME. The protocols for this study were reviewed and approved by institutional review boards at each study center. Children provided written assent; parent(s) or guardians provided written consent for the study. All scans were obtained and analyzed at Yale University.

### 2.1 Participants

The PT neurocognitive cohort consisted of the 437 surviving former PT participants, enrolled in the follow-up MRI component of the Multicenter Randomized Indomethacin Intraventricular Hemorrhage Prevention Trial (Ment et al., 1994). The PT participants all weighed between 600 and 1250 g at birth. These participants were evaluated at ages 16 and 18 with neurobehavioral testing.

At age 16, 161 PTs were included in the analysis of Child Behavior Checklist (CBCL) (Achenbach and Ruffle, 2000) testing. 276 participants were excluded from analysis: 100 were lost to follow-up, 86 had evidence of intraventricular hemorrhage, low-pressure ventriculomegaly, and/or periventricular leukomalacia (based on ultrasound within the first five postnatal days), and 89 were excluded due to having incomplete testing on the Weschler Intelligence Scale for Children, Third Edition (WISC) (Wechsler, 1991), CBCL or demographic questionnaires. One PT who scored >3 interquartile range above the third quartile on any subscale of the CBCL was labeled as an outlier and excluded from analysis.

A subset of participants was tested with the Youth Self Report (YSR) (Achenbach and Ruffle, 2000) at age 16. 45 PTs were included in this analysis. From the full cohort of PTs, 100 were excluded due to being lost to follow up, 86 were excluded due to having evidence of perinatal brain injury, and 193 subjects were excluded because they were not tested with the YSR. An additional 3 subjects were excluded due to having incomplete WISC or demographic questionnaires. 10 subjects who scored >3 interquartile range below the first quartile or above the third quartile on any of the measures of the YSR were labeled as outliers and excluded from the analysis.

At age 18, 190 PTs in total were included in the analysis. 245 participants were excluded from analysis: 143 were lost to follow-up, 75 had evidence of intraventricular hemorrhage, low-pressure ventriculomegaly, and/or periventricular leukomalacia, and 28 were excluded due to having incomplete testing on the WISC, Vineland Adaptive Behavior Scales, Second Edition (VABS) (Sparrow et al., 2005) or demographic questionnaires. There were no PTs excluded due to outlier scores on the WISC or VABS.

Term (T) control participants were matched to the PT participants with respect to age, sex, and zip code, as a proxy for socio-economic status. At age 16, 66 Ts were included in the CBCL analysis, after excluding 27 participants for missing data and 9 for outlier scores. Additionally at age 16, 56 Ts were included in the YSR analysis, after excluding 41 participants for missing data and 5 for outlier scores. At age 18, 71 Ts were included in the VABS analysis, after excluding 10 participants for missing data and 14 participants for outlier scores.

PT and T participants (*n* = 47) from the neurocognitive cohort were recruited for the MRI study at age 20 years. In total, 17 Ts and 19 PTs, all with complete neurocognitive data at ages 16 and 18, met data quality criteria (described below) and were included in the imaging portion of the study.

### 2.2 Neurobehavioral testing

All participants were tested with the CBCL at age 16 years and the VABS at age 18 years to assess social and emotional development. Participants also completed the Weschler Intelligence Scale for Children, Third Edition (WISC-III) at age 16 years to assess intellectual ability, from which Full-Scale IQ (FSIQ) scores were used in the analysis. A subset of participants was tested with the Youth Self Report (YSR) at age 16 years to assess social and emotional development from the participant's, rather than the parent's, point of view. T scores were used for the CBCL, YSR, and VABS.

The CBCL is a validated, parent/caregiver-completed questionnaire of child psychological development. Measures of social development included in this study included scores in the following: Social Competence, Social Problems, Anxiety Problems, Anxious/Depressed, Withdrawn, and Affect Problems. In this questionnaire, higher scores for Social Problems, Anxiety Problems, Anxious/Depressed, Withdrawn, and Affect Problems reflect poorer functioning, whereas lower scores in Social Competence reflect poorer functioning. The YSR is similar to the CBCL, but is self-administered (Achenbach, 1991). Measures from this instrument included in this study include the following: Activities and Social (subscales) and Anxious/Depressed, Withdrawn, and Social Problems (syndrome scales). DSM Affective Problems and DSM Anxiety Problems scales were also included. These DSM-oriented scales are comprised of measures consistent with DSM-5 categories (Affective Problems: dysthymia and major depressive disorder; Anxiety Problems: generalized anxiety disorder, separation anxiety, and specific phobia) as identified by experts.

The VABS is a clinician administered interview with a parent or caregiver that evaluates adaptive and maladaptive behavior in children. Measures of social development used from the VABS included scores in the following domains: Adaptive Behavior, Socialization, Interpersonal Relationships, Play and Leisure Time, and Coping Skills. A higher score reflects a better level of function in that domain. The latter three scales are sub of the “socialization” scale in the VABS.

### 2.3 Image parameters

Participants were scanned in a Siemens 3 T Tim Trio scanner as previously described. After a first localizing scan, a high-resolution 3D volume was collected using a magnetization prepared rapid gradient echo (MPRAGE) sequence (176 contiguous sagittal slices, slice thickness 1 mm, matrix size 192 × 192, FoV = 256 mm, TR = 2530 ms, TE = 2.77 ms, flip angle = 7°). Next, a T1-weighted anatomical scan (TR = 300 ms, TE = 2.55 ms, FoV = 220 mm, matrix size 256 × 256, thickness = 6 mm thick, gap = 1 mm) was collected with 25 AC-PC aligned axial-oblique slices. After these structural images, acquisition of functional data began in the same slice locations as the axial-oblique T1-weighted 2D Flash image. Functional images were acquired using a T2* sensitive gradient-recalled single shot echo-planar pulse sequence (TR = 1550 ms, TE = 30 ms, flip angle = 80, Bandwidth = 2056 Hz/pixel, 64*64 matrix, field of view: 220 mm × 220 mm, interleaved acquisition). Two functional runs consisted of 190 volumes (5 min scan length) with the first four volumes discarded to allow the magnetization to reach the steady-state.

### 2.4 Common space registration

First, anatomical images were skull stripped using FSL (https://fsl.fmrib.ox.ac.uk/fsl/) and any remaining non-brain tissue was manually removed. All further analyses was performed using BioImage Suite (Joshi et al., 2011) unless otherwise specified. Anatomical images were linearly aligned to the MNI brain using a 12 parameter affine registration by maximizing the normalized mutual information between images. Next, anatomical images were non-linearly registered to an evolving group average template in an iterative fashion using a previously validated algorithm (Scheinost et al., 2017a). This algorithm iterates between estimating a local transformation to align individual brains to a group average template and creating a new group average template based on the previous transformations. The local transformation was modeled using a free-form deformation parameterized by cubic B-splines. This transformation deforms an object by manipulating an underlying mesh of control points. The deformation for voxels in between control points was interpolated using B-splines to form a continuous deformation field. Positions of control points were optimized using a conjugate gradient descent to maximize the normalized mutual information between the template and individual brains. After each iteration, the quality of the local transformation was improved by increasing the number of control points and decreasing the spacing between control points to capture a more precise alignment. A total of 5 iterations were performed with decreasing control point spacings of 15 mm, 10 mm, 5 mm, 2.5, and 1.25 mm. To help prevent local minimums during optimization, a multi-resolution approach was used with three resolution levels at each iteration. The functional data were linearly registered to the 2D Flash image. The 2D Flash image was linearly registered to the MPRAGE image. All transformation pairs were calculated independently and combined into a single transform, warping the single participant results into common space. This single transformation allows the individual participant images to be transformed to the common space with only one transformation, thereby reducing interpolation error.

### 2.5 Connectivity processing

Images were slice time and motion corrected using SPM8 (http://www.fil.ion.ucl.ac.uk/spm/). Several covariates of no interest were regressed from the data, including linear and quadratic drifts, mean cerebral-spinal-fluid (CSF) signal, mean white-matter signal, and mean global signal. For additional control of possible motion-related confounds, a 24-parameter motion model (including six rigid-body motion parameters, six temporal derivatives, and these terms squared) was regressed from the data. The functional data were temporally smoothed with a Gaussian filter (approximate cutoff frequency = 0.12 Hz). A gray matter mask was applied to the data, so only voxels in the gray matter were used in further calculations.

### 2.6 Motion analysis

As group differences in motion have been shown to confound connectivity studies, we calculated the average frame-to-frame displacement for each participant's data. In line with current reports, we detected no significant difference between PTs and Ts (PTs: motion = −0.14 ± 0.07; Ts: motion = 0.11 ± 0.04; *p* > .05). To ensure that high motion time points did not influence our results, data were censored by removing all time points with a frame-to-frame displacement greater 0.20 mm of motion prior to seed connectivity. On average, we retained 85% and 90% of the data for PTs and terms, respectfully. The number of frames retained did not differ by groups (PTs: 317 ± 75, Ts: 338 ± 34, *p* = .28). The minimum amount of data for any participants was 5 min, 48 s. We did not exclude any participants after cencoring.

### 2.7 Amygdala seed connectivity

**Fig. 1:**
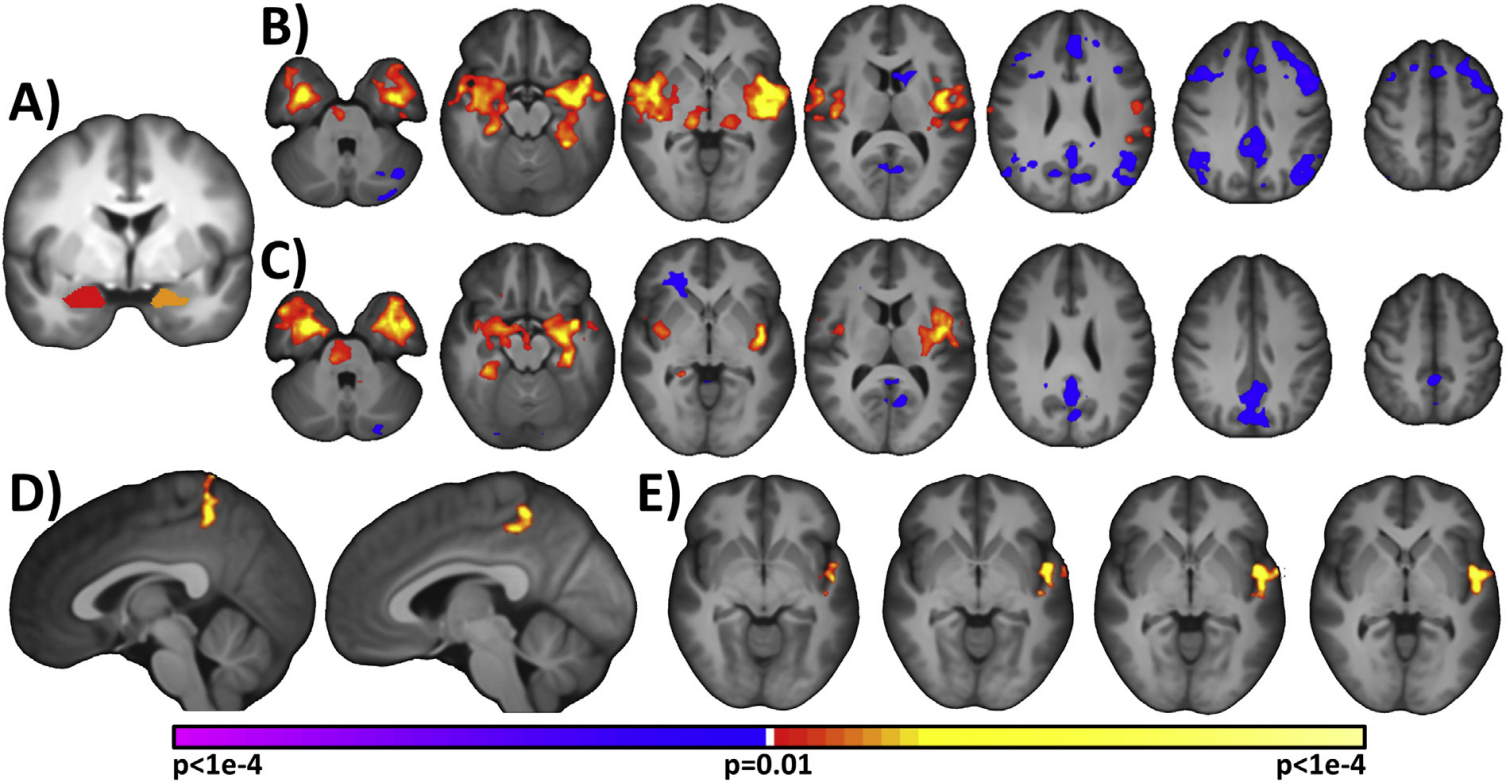
Amygdala seed connectivity. A) The bilateral amygdala seed is shown in orange and red. The amygdala connectivity based on the bilateral seed is shown B) for preterms and C) for terms. For both groups, the amygdala is connected positively to the insula, temporal region/left superior temporal gyrus, and hippocampus. Regions negatively connected to the amygdala include the posterior cingulate (PCC) for both study groups, and dorsolateral prefrontal cortex, medial prefrontal cortex, and lateral parietal cortex for PTs. D) For PTs compared to Ts, the amygdala showed significantly increased connectivity to a region in the parietal lobe that included the left precuneus and bilateral PCC. E) Additionally, the amygdala showed significantly increased positive connectivity to the left superior temporal gyrus. Images are thresholded at *p* < .01, corrected. Slices are shown in radiological convention.

A seed comprised of the bilateral amygdala was defined for the connectivity analyses (shown in Fig. 1) on the reference brain and transformed back (via the inverse of the transforms described above) into individual participant space. To account for possible drop-out effect, participants with poor amygdala coverage in the fMRI scans were excluded (6 PT and 4 T were excluded from the analysis based on this criteria; see supplemental material for more details). The time course of the reference region in a given participant was then computed as the average time course across all voxels in the reference region. This time course was correlated with the time course for every other voxel in gray matter to create a map of r-values, reflecting seed-to-whole-brain connectivity. These r-values were transformed to z-values using Fisher's transform, yielding a map representing the strength of correlation with the seed for each participant. Finally, the connectivity maps were smoothed with a 6 mm full width half maximum Gaussian kernel.

### 2.8 Statistical analyses

Demographic data were analyzed using Fisher's exact test for categorical variables and *t-*test for continuous variables. Linear regression was used to compare neurodevelopmental outcomes between PTs and Ts, with covariate adjustment for age at administration, sex, race, maternal education status, instrument respondent, and FSIQ category (≤70 or > 70). FSIQ was not added as covariate as an approximately difference inFSIQ is an intrinsic property of PTs as a whole. Controlling or matching forFSIQ in this case can lead to Lord's Paradox as the groups are no longer representative of the population as a whole (e.g. higherFSIQ PTs or lowerFSIQ Ts) (Miller and Chapman, 2001). Nevertheless, we expected significant cognitive impairment could be associated with lower social scores. While PTs are at greater risk of significant impairment, it does not define PTs as a population (for example, PT adults achieved similar educational levels as Ts (Saigal et al., 2016a)). As such, we used IQ < 70 as a measure of significant cognitive impairment and controlled for this variable. Additionally, removing all participants with an IQ < 70 from analysis or removing this covariate did not change our results. Significance was assessed at *p* < .05.

Imaging data were analyzed using voxels *t*-tests. Significance was assessed at a cluster-level threshold of *p* < .01 family-wise error correction for between group comparisons. All maps were corrected for multiple comparisons across gray matter using cluster-level correction estimated via Monte Carlo simulations. AFNI's 3dClustSim (version 16.3.05 which fixed the 3dClustSim “bug”) was used to estimate a cluster size of 1701 mm^3^ using 10,000 iterations, an initial *p*-value threshold of 0.01, the gray matter mask using in preprocessing, and smoothness values estimated from the residuals using 3dFWHMx.

Exploratory analyses were performed in the sub-cohort of imaged participants to assess the association between functional connectivity and behavior using Pearson's correlation coefficients. We restricted our analysis to only brain regions and social behavior scores that differed significantly between PTs and Ts in the full behavioral cohort. Additionally, associations were tested within the PT and T groups separately in order to minimize bias. The significance level was *p* < .05.

## 3 Results

### 3.1 Demographic characteristics

Demographic data for the PTs and Ts for the 16, 18 and 20-year visits are shown in Table 1. The PTs and Ts included in both cohorts were similar in sex, race, and maternal education level. There was a significant difference in the percentage of participants included in the 16-year cohort who had an IQ < 70. At age 16, there was a statistically significant difference in age between PTs and Ts at time of neurodevelopmental testing. However, as the difference in age was approximately one month, it is unlikely to account for group differences in neurodevelopmental test scores. There was no difference in the age at scan for PTs and Ts included in the imaged sub-cohort. There were no significant difference between the PT neurodevelopmental and PT imaging cohorts, suggesting the imaging cohort is representative of the full neurodevelopmental cohort.

**Table 1:** Demographic data for study participants in the neurodevelopmental cohorts.

	Neurodevelopmental cohort											
	CBCL			YSR			VABS			Imaged		
	16 year T (*n* = 66)	16 year PT (*n* = 161)	p	16 year T (*n* = 56)	16 year PT (*n* = 45)	p	18 year T (*n* = 71)	18 year PT (*n* = 190)	p	20 year T (*n* = 17)	20 year PT (*n* = 19)	**p**
Female n (%)	40 (53%)	77 (48%)	0.41	35 (55%)	28 (51%)	0.62	45 (52%)	87 (46%)	0.26	7 (41%)	9 (47%)	0.75
White n (%)	50 (67%)	106 (65%)	0.85	40 (63%)	26 (47%)	0.07	63 **(**74%)	143 **(**75%)	0.89	12 (71%)	16 (84%)	0.43
Caregiver education <high school^a^, n (%)	4 (5%)	13 (8%)	0.46	4 (6%)	6 (11%)	0.38	4 (5%)	20 (10%)	0.12	1 (6)	2 (11%)	1.0
IQ < 70, n (%)	2 (3%)	18 (11%)	0.03	0 (0%)	7 (13%)	0.004	2 (2%)	15 (8%)	0.08	1 (6.67%)	0 (0%)	0.49
IQ, M ± SD	102.8 ± 16.5	89.5 ± 17	<0.001	102.8 ± 15	91.1 ± 17	<0.001	103.8 ± 16	91.6 ± 16	<0.001	99.4 ± 17	93.1 ± 10	0.20
Gestational age (weeks), M ± SD	–	28.3 ± 2	–	–	28.5 ± 2	–	–	28.1 ± 2	–	–	28 ± 1.8	–
Birthweight (grams), M ± SD	–	969.0 ± 172	–	–	952.9 ± 184	–	–	960.7 ± 181	–	–	957 ± 171	–
Age at questionnaire/scan (years), M ± SD	16.2 ± 0.3	16.1 ± 0.2	0.01	16.2 ± 0.3	16.0 ± 0.1	<0.001	18.1 ± 0.9	18.4 ± 1.4	0.09	19.9 ± 1	19.9 ± 1	0.93

a For 16-year-olds, this reflects maternal education, whereas for 18-year-olds, this reflects the education of the subject's caregiver (mother or other caregiver).

### 3.2 Behavioral analysis

At age 16, when the PTs were compared to the Ts in parent-reported behavioral scores, PTs had significantly lower (worse) scores in Social Competence and significantly higher (worse) scores in Anxious/Depressed, Anxiety Problems, Withdrawn/Depressed, and Affect Problems (See Table 2) based on the CBCL. At age 18, again in the parent-reported analysis, PTs had significantly lower (worse) scores in Socialization, Interpersonal, and Play and Leisure (see Table 2) based on the VABS. Interestingly, in the child-reported analysis (YSR), PTs only had significantly lower (better) scores in the DSM Affective Problems scale (Table 3).

**Table 2:** Social and emotional behavior scores (parent reported).

	Full cohort			Imaged sub-cohort		
Behavioral domains	16 T (n = 66)	16PT (n = 161)	p	16 T (n = 17)	16PT (*n* = 18)	p*
Respondent other than self, n (%)	0 (0%)	0 (0%)	1.00	0 (0%)	0 (0%)	1.00
Social Competence	51.91 ± 8.49	45.57 ± 9.15	**0.002**	50.8 ± 10	43.3 ± 9.2	0.03
Social Problems	51.42 ± 2.87	54.59 ± 6.37	0.065	54.2 ± 6.6	54.7 ± 8	0.83
Anxious/Depressed	50.89 ± 1.64	53.81 ± 5.57	**0.001**	52.5 ± 4.6	55.6 ± 7.1	0.14
Anxiety Problems	50.86 ± 1.82	54.28 ± 5.91	**<0.001**	52.5 ± 5.1	55.2 ± 6.0	0.17
Withdrawn/Depressed	52.21 ± 3.29	56.27 ± 7.68	**0.003**	54.3 ± 6.1	56.2 ± 6.8	0.38
Affect Problems	51.83 ± 3.26	55.24 ± 7.16	**0.014**	55.4 ± 6.8	56.6 ± 9.2	0.68

	18 T (*n* = 71)	18PT (n = 190)	p	18 T (n = 17)	18PT (n = 19)	p*
Respondent other than biological mother, n (%)	8 (11%)	31 (16%)	0.31	1 (6%)	2 (11%)	1.00
Adaptive Behavior	103.83 ± 13.18	95.28 ± 17.46	0.097	102.7 ± 17	90.3 ± 14	0.02
Socialization	108.08 ± 10.34	98.12 ± 15.44	**0.010**	105.7 ± 14	97.0 ± 14	0.07
Interpersonal	16.04 ± 2.40	14.32 ± 2.86	**0.029**	15.7 ± 2.4	14.7 ± 2.8	0.25
Play and Leisure	16.59 ± 1.09	14.61 ± 3.01	**<0.001**	15.4 ± 2.6	15.1 ± 2.7	0.69
Coping	16.30 ± 2.19	15.24 ± 2.90	0.453	16.6 ± 2.5	14.2 ± 2.9	0.01

Controlling for sex, race, caregiver education, age at time of response, respondent, and full IQ for full cohort only.

Scores are Mean ± SD.

p* = imaged subgroup, PT compared to T.

Bolded values are significant at p < 0.05.

**Table 3:** Social and emotional behavior scores (child reported).

	Full cohort		
Behavioral domains	16 T (n = 56)	16PT (n = 45)	p
Respondent other than self, n (%)	0 (0%)	0 (0%)	1.00
Activities	48.28 ± 10.40	48.09 ± 11.05	0.42
Social	52.84 ± 7.82	49.38 ± 9.41	0.07
Anxious/depressed	52.21 ± 4.15	52.27 ± 3.48	0.62
Withdrawn	52.91 ± 4.24	53.20 ± 5.07	0.86
Social problems	53.43 ± 4.63	51.98 ± 2.94	0.13
DSM scale: affective problems	52.86 ± 4.26	51.47 ± 2.10	**0.04**
DSM scale: anxiety problems	52.43 ± 4.02	52.62 ± 3.87	0.54

Controlling for sex, race, caregiver education, age at time of response, respondent, and full IQ.

Scores are Mean ± SD.

In the sub-cohort consisting of only participants with imaging data at age 20, PTs had lower (worse) scores in Social Competence at age 16 and in Adaptive Behavior and Coping at age 18 (see Table 2). Behavioral scores for the PTs and Ts included in the imaging sub-cohort were not significantly different from behavioral score in the full cohort.

In Table S1, we present additional analyses without excluding any participants with outlying values.

### 3.3 Amygdala seed connectivity analysis

For both PTs (Fig. 1B) and Ts (Fig. 1C), regions positively connected to the amygdala include the insula, temporal region/left superior temporal gyrus, and hippocampus. Regions negatively connected to the amygdala include the posterior cingulate (PCC) for both study groups, and dorsal lateral prefrontal cortex, medial prefrontal cortex, and lateral parietal cortex for PTs. For PTs compared to Ts, the amygdala showed significantly increased connectivity to a region in the parietal lobe that included the left precuneus and bilateral PCC (Fig. 1D). Additionally, the amygdala showed significantly increased positive connectivity to the left superior temporal gyrus (LSTG) (Fig. 1E).

### 3.4 Exploratory analysis

Combined, PT and T amygdala-PCC connectivity was significantly negatively correlated with Social Competence on the CBCL and Socialization on the VABS (Fig. S1 and Table 4). Independently, PTs and Ts showed negative fit lines between both measures and amygdala-PCC connectivity, suggesting that group differences in the measures or connectivity were not responsible for the observed correlation. However, these associations did not reach significance. Additionally, while Anxious/Depressed, Withdrawn, and Affect Problems were significantly correlated with amygdala-PCC connectivity, these correlations were driven by high leverage points. No significant correlations between socialization measures and amygdala-LSTG connectivity were observed (Table 4).

**Table 4:** Correlation between amygdala connectivity and social and emotional behavior scores.

Amygdala-PCC			Amygdala-L STG		
Domains measured at 16 years by CBCL	Rho	P	Domains measured at 16 years by CBCL	Rho	P
Social competence (n = 35)	−0.37	0.03	Social competence (n = 35)	−0.31	0.07
Anxious/depressed (n = 32)	0.38	0.02	Anxious/depressed (n = 32)	0.23	0.20
Anxiety problems (n = 33)	0.27	0.13	Anxiety problems (*n* = 33)	0.29	0.10
Withdrawn (n = 35)	0.35	0.04	Withdrawn (n = 35)	0.02	0.92
Affect Problems (n = 35)	0.34	0.04	Affect problems (n = 35)	0.07	0.70

Domains measured at 18 years by VABS	Rho	P	Domains measured at 18 years by VABS	Rho	P
Socialization (n = 36)	−0.42	0.01	Socialization (n = 36)	−0.19	0.26
Play and leisure (n = 36)	−0.22	0.20	Play and leisure (n = 36)	−0.03	0.86
Interpersonal (n = 36)	−0.19	0.26	Interpersonal (n = 36)	−0.09	0.62

## 4 Discussion

Using neurobehavioral testing and resting state fMRI, we demonstrate that very preterm-born adolescents are more vulnerable to social impairments than their term-born peers in the view of their parents, that amygdala connectivity is altered for those prematurely-born, and that behavioral markers of social functioning correlate with altered amygdala-PCC connectivity. As our cohort of PTs does not include participants who suffered any form of perinatal neurological impairment, this increased social impairment may be attributable to prematurity rather than to other neurologic injury. The correlation between amygdala hyperconnectivity and measures of social functioning in PT adolescents suggests one possible neural underpinning for the PT social phenotype, which consists of a constellation of symptoms including increased social difficulties, heightened levels of anxiety and depression, decreased extroversion, and poor self-esteem that has been previously described.(Allin et al., 2006; Eryigit-Madzwamuse et al., 2015; Johnson and Marlow, 2014; Saigal et al., 2016b).

Compared to term-born peers, PTs in this cohort show decreased parent-reported social competence and socialization, which are composite measures of social skills including interpersonal relationships, involvement in activities, and coping skills in social situations. PTs in this cohort also show increased anxiety, depression, and affect problems, which is consistent with previous work showing that individuals who are born preterm are at higher risk for psychiatric disorders such as anxiety, depression, and phobias beginning in early school age, (Treyvaud et al., 2013) and persisting into adolescence and young adulthood (Burnett et al., 2011; Johnson and Marlow, 2011, Johnson and Marlow, 2014). These findings echo previous descriptions of social and emotional behavior in PTs (Montagna and Nosarti, 2016) providing further evidence that social impairment seen in PT children without perinatal neurologic injury persists into adolescence.

In contrast, PTs in this study did not show any difference in social competence or in anxiety and depression when measured by child report, and in fact scored significantly lower on the DSM: Affective Problems scale on the YSR, which consists of measures that are consistent with dysthymia and major depressive disorder (Achenbach, 1991). This discordance between parent and child report of characteristics of PT children and adolescents has been previously described (Dahl et al., 2006; Dinesen and Greisen, 2001). Our results support the notion that PT do not view themselves as impaired in social functioning or as having increased anxiety or depression compared to term born peers. This notion could be for several reasons. It is possible that PTs do not value the same level of social interaction as Ts, and therefore don't perceive altered social functioning where their parents do. It is also possible that PTs view themselves as on par with T born peers in terms of social development, whereas parents perceive a difference. Further study, including more objective measures of social functioning, will be necessary to fully explore this difference between parent and child reports of PT social functioning.

Our data provide further evidence that alterations in PT amygdala connectivity are a continuum across the lifespan. The amygdala is among those regions that experience the earliest prenatal structural and functional growth (Ulfig et al., 2003) and is a major hub of the “social brain” (Adolphs, 2009; Bickart et al., 2014; Shaw et al., 2005). Amygdala functional connectivity is altered in PT-born neonates (Rogers et al., 2017; Scheinost et al., 2016) as well as in PT-born adults (Papini et al., 2016). Although early adolescence represents a period of amygdala connectivity changes in typically developing controls, (Gabard-Durnam et al., 2014) to the best of our knowledge, there are no published studies of amygdala connectivity in PT at this age. However, volumetric studies demonstrate decreased amygdala volumes at age 8 years in PT compared to T controls (Peterson et al., 2000). The overall pattern of amygdala connectivity in PTs in this study was similar to previously described amygdala connectivity in healthy adults (Roy et al., 2009). Nevertheless, the PTs have decreased negative connectivity from the amygdala to the left precuneus and bilateral PCC and increased positive connectivity to the left STG when compared to term-born peers. Both are areas that have previously been implicated in social and emotional behavior in other disorders (Allison et al., 2000; Hahn et al., 2011). These findings are similar to connectivity differences found in a separate cohort of PT-born adults at age 30 (Papini et al., 2016).

Connectivity between the amygdala and PCC negatively correlates with measures of social functioning in a cohort of combined PTs and Ts. Hyperconnectivity of the amygdala to the PCC has been associated with childhood anxiety disorders (Hamm et al., 2014) and altered amygdala-PCC connectivity has been associated with social anxiety disorder in adults (Hahn et al., 2011; Rabany et al., 2017). Together, these studies suggest that the association between behavior and amygdala-PCC connectivity is not specific to PTs, and that hyper-connectivity in this circuit is related generally to social and emotional behavioral problems.

The posterior cingulate cortex is part of the default mode network (DMN) (see for a (Buckner et al., 2008) review) and is also thought to play a role in social cognition, (Laird et al., 2011) in particular in evaluating others' mental states (Saxe and Powell, 2006) and in emotion processing (Wright et al., 2008). As impaired emotion recognition contributes to decreased social competence, (Schultz et al., 2001) this may be a factor underlying the relationship between altered amygdala connectivity and social impairments.

Our study has several strengths: we provide further evidence of increased social impairments along with anxiety and depression in a large cohort of PTs in adolescence and demonstrate a negative correlation between social functioning and amygdala-PCC connectivity in participants with previous clinically unremarkable imaging. These data provide an evaluation of the relationship between the PT social functioning and amygdala connectivity during adolescence, a time of significant change in both social demands and neural connectivity. Furthermore, the participants included in this analysis had no history of neurologic injury, suggesting that alterations in connectivity and function are due to prematurity rather than prior injury.

There are several limitations to this analysis. First, we acknowledge that there are significant differences inFSIQ score between PTs and Ts in our large neurobehavioral cohort, consistent with existing literature (Allin et al., 2008). It is possible that lowerFSIQ may predispose the PTs to increased social impairments compared to typically developing term controls; likewise, it may alter the PTs' self-perception of this condition. We did, however, control forFSIQ in this analysis, and found that the differences in social behavior between PT and T were present after controlling for FSIQ. Second, while our imaging data is a strength of this study, the size of our imaging cohort was limited. As such, we were not able to adequately analyze the imaging-behavior correlations for PTs and Ts separately; nor were we able to control for demographic covariates. Third, while our findings demonstrate increased social impairment among PT, it is non-specific and could be due to impairments in different functional domains. Given the format of the instruments we used to evaluate social functioning, we are unable to parse out the specific mechanisms leading to this social impairment. Finally, we do not have perinatal data about other factors that may impact long term cortical development and neurodevelopmental outcomes. For example, prenatal exposure to maternal stress impacts amygdala functional connectivity (Scheinost et al., 2017b) and exposure to increased painful procedures in the neonatal period alters brain architecture and increases incidence of internalizing behaviors in PT born children.(Ranger et al., 2013; Ranger et al., 2014; Vinall and Grunau, 2014). Therefore, it will be important to re-examine the relationship between amygdala connectivity and social impairment in groups of PT with differing levels of pre- and perinatal stress in order to more accurately risk-stratify this population.

As survival continues to improve for prematurely born neonates, it is increasingly important to accurately determine risk for adverse neurodevelopmental outcomes and develop interventions to mitigate these morbidities. Adolescence is a time of major social and emotional changes, including increased social pressure from peers, emerging independence from parents, and changing interpersonal relationships, (McLaughlin et al., 2015) and our results affirm that PTs continue to experience significant social and emotional difficulties during this stage of life. Future work should interrogate the developmental trajectories of altered amygdala connectivity and social impairment in PT to develop interventions that may be successful in decreasing these behavioral problems.

## Appendix A Supplementary data

Supplementary material Image 1
