# Supplementary material for: Amygdala functional connectivity is associated with social impairments in preterm born young adults
Source: Neuroimage Clin. 2018 Dec 3;21:101626. doi: 10.1016/j.nicl.2018.101626 (PMC6413301; doi:10.1016/j.nicl.2018.101626)
Supplement: Supplementary file 1 — Supplementary material [file mmc1.docx]

Supplemental methods:

Exclusion of subjects based on amygdala coverage: First, we warped the amygdala seed as defined on the MNI template into single subject fMRI space. Next, we computed a mask of voxel containing fMRI signal using AFNI’s 3dAutomask program. (This program automatically creates a brain mask based on the distributions of image intensities). Then, we multiplied the warped amygdala seed and the automatically generated brain mask to create a third mask containing only voxels in the amygdala that contained fMRI signal. Finally, the overlap was computed as the percent of amygdala voxels remaining in the third mask compared to the original warped amygdala seeds. An overlap of less than 30% was chosen for exclusion as this overlap corresponded to a volume of approximate equal volume of 5 mm radius seed commonly used in the field (~500mm^3). (Note the volume corresponding to 30% overlap was slightly different for each subject due to the nonlinear warping accounting for brain size, but on average 30% overlaps corresponded to the same volume as a 5mm radius sphere).

Supplemental results

As we limited our correlation analysis between imaging and behavioral data to only regions with group differences, we did not test if amygdala-dlPFC connectivity correlated with social behavior. Nevertheless, given the previous literature on amygdala-dlPFC connectivity, we proceeded to explore these correlations. Using a 5 mm radius sphere in the dlPFC based on the PT’s group mean amygdala seed map, we extracted amygdala-dlPFC connectivity for further analysis. The PTs exhibited a trend for lower connectivity (p=0.11) compared to the terms. We did not observe any correlations between amygdala-dlPFC connectivity and our social measures for the PTs or Ts independently or for the groups combined. These negative results may be that we lack power or that the spread of our social measures are not sufficient to detect association related to individual differences in socialization. Additionally, imaging was performed at 20 years of age while the social measures were assessed at 16 and 18 years of age. It is possible that measures collected at age 20 may better relate to amygdala-dlPFC connectivity.

Supplemental results:

TABLE S1. Social and Emotional Behavior Scores in full cohort, without excluding outliers

|  | **Full Cohort** | | |
| --- | --- | --- | --- |
| **CBCL** | **16T (n=75)** | **16PT (n=162)** | **p** |
| Respondent other than self, n (%) | 0 (0%) | 1 (0.1%) | 0.5 |
| Social Competence | 50.87 ± 9.11 | 45.58 ± 9.12 | **0.02** |
| Social Problems | 52.81 ± 5.06 | 54.65 ± 6.39 | 0.7 |
| Anxious/Depressed | 51.97 ± 3.78 | 53.99 ± 5.98 | **0.05** |
| Anxiety Problems | 52.33 ± 4.84 | 54.40 ± 6.08 | 0.08 |
| Withdrawn/Depressed | 53.68 ± 6.40 | 56.38 ± 7.79 | 0.15 |
| Affect Problems | 54.16 ± 5.42 | 55.39 ± 7.38 | 0.27 |
| **YSR** | **16T (n=61)** | **16PT (n=52)** | **p** |
| Respondent other than self, n (%) | 0 (0%) | 0 (0%) | 1 |
| Activities | 48.03 ± 10.34 | 47.90 ± 10.82 | 0.32 |
| Social | 52.54 ± 8.21 | 49.60 ± 9.69 | 0.14 |
| Anxious/Depressed | 53.48 ± 5.94 | 53.92 ± 5.61 | 0.93 |
| Withdrawn | 53.69 ± 5.04 | 55.00 ± 6.96 | 0.55 |
| Social Problems | 54.25 ± 5.77 | 53.42 ± 5.69 | 0.56 |
| DSM scale: affective problems | 53.70 ± 5.29 | 53.35 ± 5.27 | 0.42 |
| DSM scale: anxiety problems | 53.62 ± 5.70 | 54.29 ± 5.96 | 0.88 |
| **VABS** | **18T (n= 85)** | **18PT (n=190)** | **p** |
| Respondent other than biological mother, n (%) | 11 (13%) | 30 (16%) | 0.54 |
| Adaptive Behavior | 100.18 ± 14.91 | 95.28 ± 17.46 | 0.73 |
| Socialization | 103.54 ± 14.4 | 98.12 ± 15.44 | 0.53 |
| Interpersonal | 15.32 ± 2.86 | 14.32 ± 2.86 | 0.65 |
| Play and Leisure | 15.58 ± 2.54 | 14.61 ± 3.01 | 0.24 |
| Coping | 15.86 ± 2.54 | 15.24 ± 2.90 | 0.87 |

Controlling for sex, race, caregiver education, age at time of response, respondent, and full IQ.

Scores are Mean±SD

**
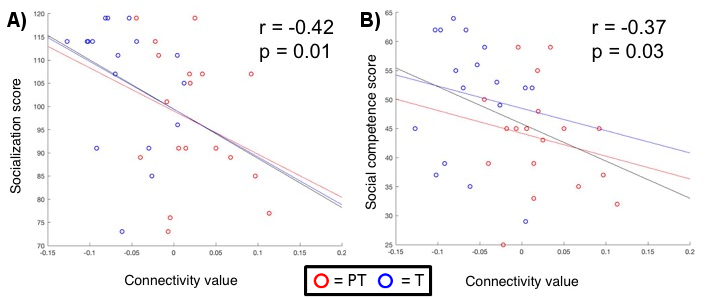
**

**Figure S1: Association between amygdala-PCC connectivity and social vulnerability.** Combined, PT and T amygdala-PCC connectivity was significantly negatively correlated with **A)** Socialization on the VABS and **B)** Social Competence on the CBCL. Independently, PTs and Ts showed negative fit lines between both measures (Socialization and Social Competence) and amygdala-PCC connectivity, suggesting that group differences in the measures of connectivity were not responsible for the observed correlation. Best fit line for PTs is shown in red, for Ts is shown in blue, and for both groups combine is shown in grey.
